# Phylogenetic Relationship Between Two Squirrel Species, *Callosciurus pygerythrus* and *Funambulus pennantii*, Inferred From Mitochondrial Cytochrome *b* Gene Sequences

**DOI:** 10.1002/vms3.70550

**Published:** 2025-08-13

**Authors:** Sanjida Abida, Md. Sirazul Islam, Zebunnahar Yasmin, Nachimuthu Senthil Kumar, Amam Zonaed Siddiki, Md. Farid Ahsan

**Affiliations:** ^1^ Department of Zoology University of Chittagong Chittagong Bangladesh; ^2^ Department of Pathology and Parasitology Faculty of Veterinary Medicine Chattogram Veterinary and Animal Sciences University Chattogram Bangladesh; ^3^ Department of Biotechnology Mizoram University Aizawl Mizoram India

**Keywords:** *Callosciurus pygerythrus*, cytochrome *b* gene, *Funambulus pennantii*, genetic diversity, phylogenetic relationship

## Abstract

To investigate the phylogenetic relationship between two species of squirrels, the hoary‐bellied Himalayan squirrel *Callosciurus pygerythrus* and five‐striped palm squirrel *Funambulus pennantii*, at the population level to assist conservation and management, we analysed partial mitochondrial cytochrome *b* gene sequences (1140 base pairs). The hoary‐bellied Himalayan squirrels were collected from the Chittagong University campus and the Hazarikhil Wildlife Sanctuary, Chattogram, and the five‐striped palm squirrels were collected from the Rajshahi Zoo area of Bangladesh. Blood samples were collected from anaesthetized squirrels by expert veterinarians, drawing blood from femoral veins in accordance with proper protocols and permission from respective authorities for the PCR (polymerase chain reaction) study. Mitochondrial sequences from seven individual animals were submitted to the global NCBI (National Center for Biotechnology Information) database, retrieved seven accession numbers  (MT227570, MT252006, MT227571, MT227572, MT227573, MT227574 and MT227575), and data analyses using modern bioinformatics tools were conclusive in identifying the animals. The phylogenetic relationship between the two species was inferred from the partial mitochondrial cytochrome *b* gene of 1140 bp using Mega software in maximum likelihood methods. Results reveal a close genetic relationship among all haplotypes of *C. pygerythrus* within a single clade, supported by a high bootstrap value of 61%, providing insight into its systematic position. On the other hand, three subjected haplotypes of *F. pennantii* were observed to exhibit close relatedness to another two Australian haplotypes of *F. pennantii* sourced from the GeneBank, with a bootstrap value of 87%, contributing valuable insights into its systematic position. The divergence of the two species into distinct clusters on the phylogenetic tree is attributed to substantial morphological differences and significant genetic distances. Phylogenetic analysis among all available haplotypes to date among the two genera and species provides valuable analytical perspectives about intra‐ and inter‐specific relationships and quantifies the genetic diversity between Bangladesh and other geographic populations. The study emphasizes the necessity of conducting additional research with a substantial sample size to comprehensively investigate the genetic diversity of squirrel population in Bangladesh. The study will enhance our comprehension of wildlife biodiversity and contribute to the development of natural conservation strategies for these species. The global and local status of both of the species is ‘Least Concern’.

## Graphical abstract

This study demonstrates the morphological as well as molecular study and phylogenetic relationship of two species of squirrel, the five‐striped palm squirrel and hoary‐bellied Himalayan squirrel. It also analyses the phylogenetic relationship among other squirrels based on cytochrome *b* gene of the two genera and species to assist conservation and management.

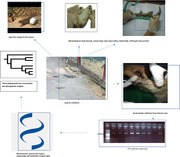

## 1 Introduction

Squirrels (Sciuridae: Rodentia) are small‐to‐medium‐sized rodents. The Sciuridae comprises 273 species under 50 genera worldwide (Hoffmann et al. 1993; Thorington et al. 2002). In Bangladesh, there are nine species of squirrels (Khan 2015; IUCN Bangladesh 2015; Khan MMH 2018); all these have been identified on the basis of morphological characteristics, which have been long practiced by biologists throughout the world since the beginning of the binomial nomenclature of species done by Linnaeus in 1758.

Species identification using molecular tools is more reliable for any new taxa than considering the external morphological study (e.g., Somaweera 2004; Crawford et al. 2010). Molecular or DNA barcoding analysis is widely applied to determine genetic family relationships, such as the phylogeny study of an organism, and scientists recommend it all over the world (e.g., Avise 2000; Hebert et al. 2003).

The utility of DNA barcoding for species identification and discovery has catalysed a concerted effort to build the global reference library (Ermakov et al. 2015) and essential tools for constructing a reliable phylogenetic tree. Identification, analysis of genetic diversity, gene mapping and evolutionary study of squirrels worldwide by the molecular method are prevalent today. As the mutation rate is prolonged in the mitochondrial gene, scientists prefer the mitochondrial gene for phylogenetic study.

The phylogenetic work on squirrels in Bangladesh based on molecular study remains unsolved. So, this study on the Cytochrome *b* gene of mitochondrial DNA was carried out to establish the phylogenetic relationship between two species of squirrels, the hoary‐bellied Himalayan squirrel *Callosciurus pygerythrus* and five‐striped palm squirrel *Funambulus pennantii* (Wroughton 1905) after morphological study to obtain better information concerning genetic diversity for their conservation and management.

## 2 Materials and Methods

### 2.1 Sample Collection Area

The samples were collected from three locations in Bangladesh spanning from January 2019 to December 2019. *C. pygerythrus* were collected from the Chittagong University campus (22.42340 N; 91.41536 E), and *F. pennantii* were collected from the Rajshahi Zoo area (24.36573 N; 88.56944 E) of Bangladesh.

### 2.2 Sample Collection and Preservation

**FIGURE 1:**
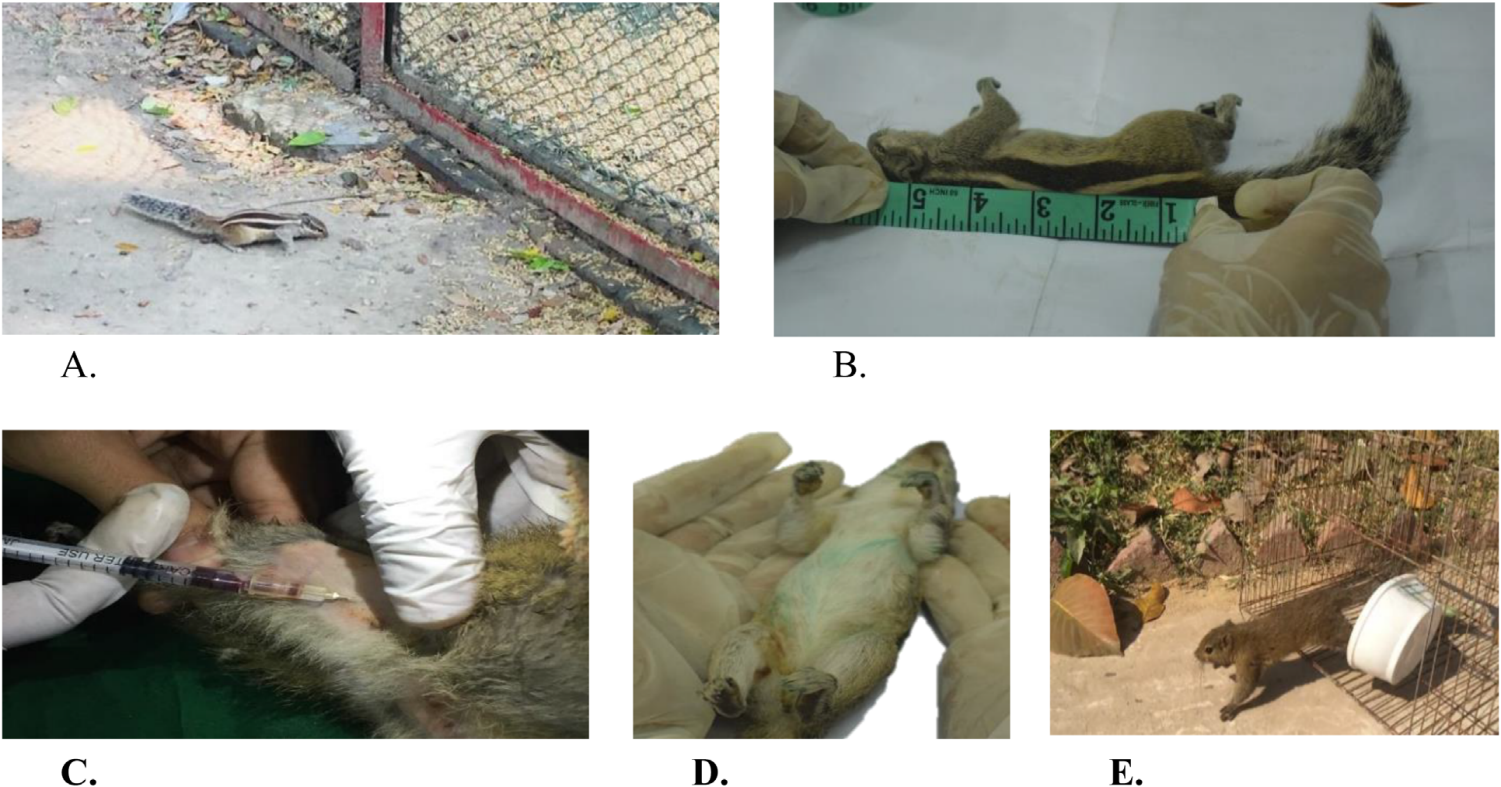
(A) Squirrel roaming outside the aviary; (B) observation of the body length for the morphological study; (C) blood sample collection; (D) dorsal view of a northern palm squirrel; (E) a released squirrel in its original habitat after study.

Squirrels are small, swift and agile animals with sharp claws. Special care was taken to handle them; that is, wearing rubber gloves during handling and holding on to the neck to avoid biting and scratching. Squirrels were trapped after selecting sites, and mice traps (28 cm × 15 cm × 15 cm) were placed near bushy places over the day and night using tomatoes and biscuits as bait. Used local mice traps were carried to the laboratory. After morphologically identifying them, features were recorded in a notebook, and the specimens were anaesthetized with diazepam and ketamine following IACUC guidelines of 2014. Then measurements for the set parameters were taken, and blood samples were collected following the respective protocol into EDTA vials. Blood was then kept at a temperature below −20°C for the study. After blood collection, the squirrels were released to their native habitats with vigilant observations of their health status (Figure 1E).

### 2.3 Morphological Identification

#### 2.3.1 Hoary‐Bellied Himalayan Squirrel

*C. pygerythrus* is a dark rufous‐brown animal, sometimes slightly speckled with dark yellowish‐brown. It is distinctive with a greyish, isabelline or pale rufous belly. The longer dorsal hairs have two light rings (Prater 1971). The species has been assessed as Least Concern category both nationally (Begum 2015) and globally (Duckwort 2016) by the IUCN. It lives in dense forests, well‐wooded villages and semi‐urban habitats with plantations bordering forests; a few pairs live in Dhaka city too (Begum 2015) (Table 1).

**TABLE 1:** Morphological study of hoary‐bellied Himalayan squirrel *Callosciurus pygerythrus* and five‐striped palm squirrel *Funambulus pennantii*.

Name	Dorsal (body colour)	Ventral (body colour)	Tail	Head–body length (cm)	Tail length (cm)	Mean HBL (cm)	Mean TL (cm)
*Callosciurus pygerythrus*	Brown **Previous record**: Olive‐brown to grey or greyish coat colour (Prater 1971)	Pale white **Previous record**: White or isabelline belly (Prater 1971; Kamruzzaman 2009)	Bushy brown long tail larger than the body	19–22.2 **Previous record**: 20 cm (Prater 1971)	20.3–22.8 **Previous record**: A little larger than body	21.28	21.74
*Funambulus pennantii*	Grizzled grey–brown colour with five conspicuous white stripes, three from neck to base of tail and the other two from fore to hind leg **Previous record**: Five stripes on back. Three median, pale, dorsal stripes flanked on each side	Creamy white **Previous record**: White belly (Chakma 2009)	Bushy tail slightly shorter than its body with long interspersed black and white hairs	15.8–17.2 **Previous record**: 13 cm (Prater 1971)	15.9–18.5 **Previous record**: Slightly larger than the body (Prater 1971)	16.67	17.3

Abbreviations: HBL, Head‐body‐length; TL, total length.

### 2.4 Five‐Striped Palm Squirrel

*F. pennantii* has five pale stripes on its back: three median, pale and dorsal stripes flanked on each side with a supplementary pale stripe. Local races are recognized on the basis of the lightness and darkness of the coat or variations in the tone of dorsal stripes (Prater 1971). The species is considered in the least concern category nationally (Jaman 2015) and globally (Nameer and Molur 2016). It inhabits the Indian peninsula from the base of the Himalayas southwards, is more common in North India, is more found in arid portions and is more commensal with man (Prater 1971). In Bangladesh, it is mostly distributed in the west of the Jamuna River (Jaman 2015) and spreading its range day by day, and it has already somehow invaded Suhrawardy Park in Dhaka (MFA personal observations) (Table 1).

### 2.5 DNA Extraction and Molecular Analysis

Total DNA was extracted using a genomic DNA extraction kit (AddBIO, South Korea). The cytochrome *b* gene was polymerase chain reaction (PCR)‐amplified using primers L14734: 5′‐GATATGAAAAACCATCGTTGTTTACAAGACCGAG‐3′ and H15910: 5′‐GATTTTTGGTTTACAAGACCGAG‐3′ previously designed by Oshida et al. (2000). The PCR thermal cycle employed was as follows: initial denaturation at 95°C for 5 min, followed by 35 cycles of amplification at 94°C for 1 min, 55°C for 1 min and 72°C for 2 min; and a final elongation at 72°C for 10 min. To remove primers and unincorporated nucleotides, the amplified products were purified using a DNA Purification kit (AddBIO, South Korea). The purified DNA samples were sent to a commercial supplier (Biotech Concern Pvt. Ltd, Bangladesh) for sequencing. The data were analysed using available bioinformatics tools, that is, MEGA 10, Bio‐edit7.2 and CLUSTALW.

### 2.6 DNA Sequencing and Phylogenetic Analysis

All available haplotypes based on mitochondrial cytochrome *b* gene were retrieved from the National Center for Biotechnology Information (NCBI) database of the two subjected genera and species as far as our knowledge to this date. Three phylogenetic trees were constructed using the maximum likelihood method. The bootstrap values were calculated using 500 replicates (Felsenstein 1985). The evolutionary distances were computed using the maximum composite likelihood method (Tamura and Nei 1993). The phylogenetic analyses were conducted using MEGA‐X (Kumar et al. 2018).

## 3 Results

### 3.1 Result of Morphological Findings

The seven squirrels (hoary‐bellied Himalayan and five‐striped palm) were identified morphologically (Table 1) by assessing their dorsal and ventral body colours and measuring their head–body length and tail length based on Prater (1971) and Menon (2003). The body length (snout to vent) of hoary‐bellied Himalayan squirrel ranged from 19 to 22.2 cm, with a mean of 21.28 cm, whereas the tail length varied from 20.3 to 22.8 cm, with an average of 21.74 cm. The body length of five‐striped palm squirrel ranged from 15.8 to 17.2 cm, with an average of 16.67 cm. The tail length ranged from 15.9 to 18.5 cm with an average of 17.3 cm. The hoary‐bellied Himalayan squirrel was identified by its dorsal olive–brown to grey coat colour and ventral white or isabelline belly and compared to its old record of identification (Prater 1971; Blanford 1891; Menon 2003).

Similarly, the five‐striped palm squirrel was also identified by its length and dorsal grizzled grey–brown colour with five conspicuous white stripes, three from neck to base of tail and the other two from fore to hind legs and ventral creamy white belly and compared to the old record of identifications (Prater 1971; Menon 2003) (Figure 1).

### 3.2 Molecular Confirmation and Data Archiving

**FIGURE 2:**
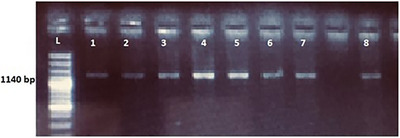
Amplified DNA fragment bands derived after PCR in 1% agarose.

After PCR, sequences were examined by agarose gel electrophoresis, and 8 of 10 samples were amplified when observed under UV‐ *trans*‐illuminator (Figure 2).

The partial sequences (1140 bp) of cytochrome *b* gene were sequenced, and the following eight sequences were submitted to the NCBI GeneBank and seven accession ID were retrieved (Table 2).

**TABLE 2:** Supplementary dataset of the accession number of National Center for Biotechnology Information (NCBI) GenBank used for phylogenetic analysis.

Sequence ID	NCBI accession	Collection date	Location of collection
Seq1	MT227570	28 January 2019	22.28.192 N 91.48 E
Seq2	MT252006	29 January 2019	22.28.192 N 91.48 E
Seq3	MT227571	20 February 2019	22.28.192 N 91.48.13 E
Seq4	MT227572	15 October 2019	24.3657.3 N 88.5694.43 E
Seq5	MT227573	15 October 2019	24.3657.3 N 88.5694.43 E
Seq6	MT227574	7 November 2019	22.42.340 N 91.41.536 E
Seq7	MT227575	14 November 2019	24.3657.3 N 88.5694.43 E

### 3.3 Phylogenetic Relationship

**FIGURE 3:**
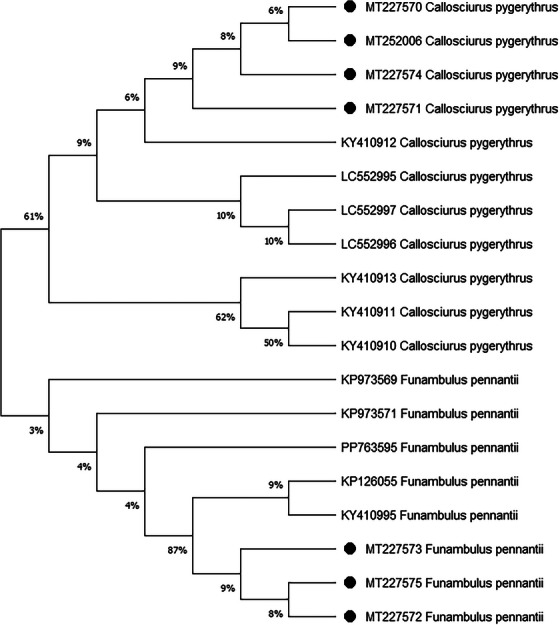
Phylogenetic comparison of mitochondrial cytochrome *b* gene sequences by ML method. The nucleotide sequences of mitochondrial cytochrome *b* gene (1140 bp) in squirrels obtained from this study (black filled circles) were compared with those of representative species variants obtained from NCBI (National Center for Biotechnology Information).

The phylogenetic analysis revealed that the sequences obtained from the seven mitochondrial cytochrome *b* genes of squirrels were closely related to other squirrels species of the world in the recent past (Figure 3). Although the study regions were diverse, the identity and similarity percentages of the sequences of the mitochondrial cytochrome *b* gene of the other squirrel species showed a close relationship among them.

### 3.4 Evolutionary Analysis of *Callosciurus pygerythrus* and *F. pennantii* by the Maximum Likelihood Method

The evolutionary history was inferred by using the maximum likelihood method and the Tamura–Nei model [1]. The tree with the highest log likelihood (−28,482.90) is shown. The percentage of trees in which the associated taxa clustered together is shown next to the branches. Initial tree(s) for the heuristic search were obtained automatically by applying the Neighbour–Join and BioNJ algorithms to a matrix of pairwise distances estimated using the Tamura–Nei model and then selecting the topology with the superior log likelihood value. The proportion of sites where at least 1 unambiguous base is present in at least 1 sequence for each descendant clade is shown next to each internal node in the tree. This analysis involved 19 nucleotide sequences. There were a total of 11,959 positions in the final dataset. Evolutionary analyses were conducted in MEGA X [2].

### 3.5 Evolutionary Analysis of the Genus *Callosciurus* by the Maximum Likelihood Method

The evolutionary history was inferred by using the maximum likelihood method and the Tamura–Nei model [1]. The tree with the highest log likelihood (−24,099.27) is shown. The percentage of trees in which the associated taxa clustered together is shown next to the branches. Initial tree(s) for the heuristic search were obtained automatically by applying the Neighbour–Join and BioNJ algorithms to a matrix of pairwise distances estimated using the Tamura–Nei model and then selecting the topology with superior log likelihood value. The proportion of sites where at least 1 unambiguous base is present in at least 1 sequence for each descendent clade is shown next to each internal node in the tree. This analysis involved 17 nucleotide sequences. There were a total of 11,497 positions in the final dataset. Evolutionary analyses were conducted in MEGA X [2].

### 3.6 Evolutionary Analysis of the Genus *Funambulus* by Maximum Likelihood Method

The evolutionary history was inferred by using the maximum likelihood method and the Tamura–Nei model [1]. The tree with the highest log likelihood (−24,099.27) is shown. The percentage of trees in which the associated taxa clustered together is shown next to the branches. Initial tree(s) for the heuristic search were obtained automatically by applying the Neighbour–Join and BioNJ algorithms to a matrix of pairwise distances estimated using the Tamura–Nei model and then selecting the topology with superior log likelihood value. The proportion of sites where at least 1 unambiguous base is present in at least 1 sequence for each descendant clade is shown next to each internal node in the tree. This analysis involved 17 nucleotide sequences. There was a total of 11,497 positions in the final dataset. Evolutionary analyses were conducted in MEGA X [2].

The phylogenetic tree (Figure 3) was constructed between two species of squirrel, *C. pygerythrus* and *F. pennantii*, using mitochondrial cytochrome *b* gene sequences, a widely used molecular marker for resolving evolutionary relationships in mammals. A total of 11 haplotypes of *C. pygerythrus* and 8 haplotypes of *F. pennantii* were analysed across 5 geographic regions: Nepal, India, Bangladesh, Myanmar and Australia. The tree shows the two species are in completely distinct evolutionary lineages.

### 3.7 Intraspecific Relationship Among all Existing Haplotype of *C. pygerythrus*

All *C. pygerythrus* haplotypes show common ancestry, supporting 61% bootstrap support.

The MT227570, MT227571, MT227574, MT252006 and KY410912 form a subclade, indicating a strong genetic relationship and recent common ancestry within Bangladeshi populations. The LC552995, LC552997 and LC552996, three haplotypes, formed a sub‐clade with bootstrap support of 10%, showing clustering of Myanmar sequences. The weak bootstrap support suggests that the close placement of the Myanmar clade near the Bangladeshi group may imply a shallow genetic divergence or recent gene flow. However, without stronger statistical support, the Myanmar haplotypes cannot yet be considered a definitively distinct lineage from the Bangladeshi cluster. Indian and Nepalese haplotypes (KY410911, KY410910 and KY410913) grouped together with a bootstrap support of 62%, confirming their close evolutionary relationship and indicating geographic cohesion between India and Nepal.

### 3.8 Intraspecific Relationship Among All Existing Haplotypes of *F. pennantii*

The phylogenetic tree based on mitochondrial cytochrome *b* gene sequences includes eight haplotypes of *F. pennantii* sampled from India, Bangladesh and Australia. The analysis reveals genetic divergence and regionally clustered clades, supported by varying levels of bootstrap confidence. Indian haplotypes (KP973569, KP973571 and KP762595) cluster together, forming the core clade of *F*. *pennantii*. The internal branches among them are supported by moderate bootstrap values, suggesting recent common ancestry within Indian populations. Their grouping reflects a genetically cohesive population with low intraspecific divergence. Bangladeshi haplotypes (MT227572, MT227573 and MT227575) form a sister clade and share strong bootstrap support (87%) with two Australian haplotypes (KP126055 and KY410995). This suggests incipient divergence between Indian and Bangladeshi populations, likely driven by geographic separation or ecological barriers.

**FIGURE 4:**
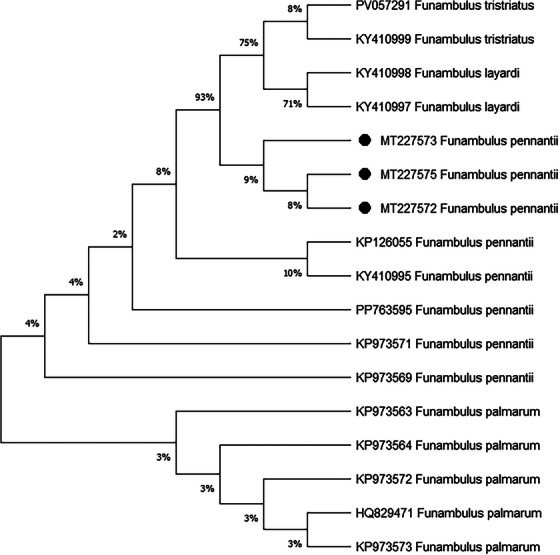
Phylogenetic comparison of mitochondrial cytochrome *b* gene sequences by ML method. The nucleotide sequences of mitochondrial cytochrome *b* gene (1140 bp) in squirrels obtained from this study (black filled circles) were compared with those of representative species variants obtained from NCBI (National Center for Biotechnology Information).

The phylogenetic tree (Figure 4) was constructed among four species of the genus *Funambulus* mitochondrial cytochrome *b* gene sequences. A total of 19 haplotypes (Figure 4) of *F. pennantii*, *Funambulus tristriatus*, *Funambulus layardi* and *Funambulus palmarum* were analysed to check the phylogenetic relationship among all available haplotypes of *Funambulus* till date. The three subjected *F. pennantii* show a close relationship with the subclade of *F. tristriatus* and *F. layardi* with 93% bootstrap support.

**FIGURE 5:**
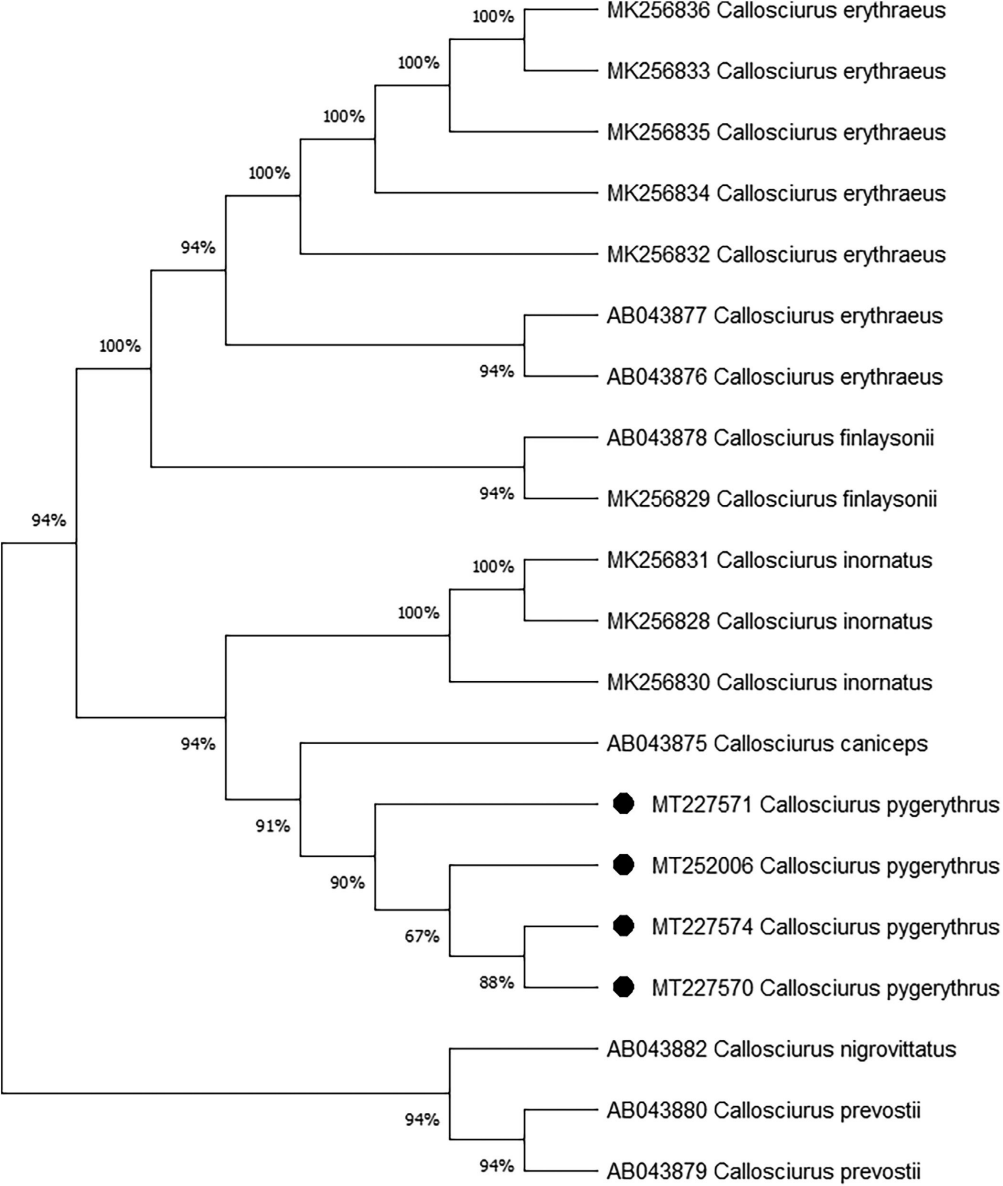
Phylogenetic comparison of mitochondrial cytochrome *b* gene sequences by ML method. The nucleotide sequences of mitochondrial cytochrome *b* gene (1140 bp) in squirrels obtained from this study (black filled circles) were compared with those of representative species variants obtained from NCBI (National Center for Biotechnology Information).

The phylogenetic tree (Figure 5) was constructed among seven species of the genus *Callosciurus* based on mitochondrial cytochrome *b* gene sequences, a widely used molecular marker for resolving evolutionary relationships in mammals. A total of 20 haplotypes (Figure 5) of *Callosciurus erythraeus, Callosciurus finlaysonii, Callosciurus inornatus, Callosciurus caniceps, C. pygerythrus, Callosciurus nigrovittatus* and *Callosciurus prevostii* were used to analyse the phylogenetic relationship among all available haplotypes of the genus *Callosciurus* in intra‐specific level to date. The species *C. pygerythrus* has a close phylogenetic relationship with two species, *Callosciurus caniceps* and *C. inornatus* by strong bootstrap support, 94%, indicating a well‐supported clade.

## 4 Discussion

In this study, we investigated the morphological characteristics, genetic diversity and phylogenetic relationships of two squirrel species: the hoary‐bellied Himalayan squirrel and the five‐striped palm squirrel. We were able to learn about the taxonomy and evolutionary history of these species by combining morphological observations, molecular confirmation using PCR and partial gene sequencing of cytochrome *b* gene and phylogenetic analysis.

Morphological examination was critical in identifying the two squirrel species. We noticed distinct differences in their dorsal and ventral body colours, as well as head–body length and tail length (Table 1). The dorsal side of the hoary‐bellied Himalayan squirrel had an olive‐brown to grey coat colour, and the ventral side had a white or isabelline belly. These morphological characteristics agreed with previous identification records (Prater 1971; Blanfor 1891; Menon 2003). The five‐striped palm squirrel, on the other hand, was a grizzled grey–brown colour with five distinct white stripes, three running from the neck to the base of the tail and two extending from the fore to the hind legs. It had a creamy white belly on its ventral side, which was consistent with previous identifications (Prater 1971; Menon 2003).

The phylogenetic analysis of the cytochrome *b* gene sequences, which included the submitted sequences as well as all available haplotype sequences obtained from NCBI database based on cytochrome *b* gene of different geographic locations. The resulting phylogenetic tree demonstrated the studied squirrel species’ close genetic relationship with other squirrel species worldwide. The presence of shared nodes with high bootstrap values indicated strong support for taxonomic clustering. Notably, the phylogenetic analysis revealed intriguing findings about genetic relationships both within species and genus. The regional structuring of mitochondrial haplotypes suggests historical geographic isolation and restricted gene flow across South Asia and Australia; the use of cytochrome *b* enables clear understanding of these intra‐ and inter‐specific relationships. As the existing haplotypes are limited in number, their position in the tree does not represent a natural lineage.

Overall, the findings of this study add to our existing understanding of the morphological characteristics and molecular identification, validating their systematic position through phylogenetic trees and the study of their genetic diversity and phylogenetic relationships of the hoary‐bellied Himalayan and five‐striped palm squirrels. Morphological identification, PCR analysis and sequencing provided valuable information for species identification and genetic characterization. The phylogenetic analysis revealed the evolutionary relationships of the two squirrel species with other squirrel species under the two genera around the world. These findings contribute to our understanding of squirrel biodiversity and evolutionary history, which can help conservation efforts and future research on these species.

## 5 Conclusion

This investigation allowed the correct identification of two squirrel species: hoary‐bellied Himalayan squirrels (*C. pygerythrus*) (Saint‐Hilaire 1832) and five‐striped palm squirrels (*F. pennantii*) (Wroughton 1905) by morphological study and mtDNA introgression. It also contributes the mtDNA sequences to the GenBank. Overall, the results of this study provide the evidence for the ability of DNA barcodes to identify most of the species of squirrels and their phylogenetic study. Our objective for this study was to use a molecular approach to test the morphological taxonomy (Hall 1981) as a hypothesis of evolutionary relationships. Finally, our study provides a better understanding of the genetic variation of those two squirrel species and found their paraphyletic relationship with other existing recorded species.

## Author Contributions

**Sanjida Abida**: sample collection, methodology, morphological and molecular investigation, data analysis, writing original draft, funding acquisition. **Md. Sirazul Islam**: sample collection, NCBI data submission, phylogenetic analysis, draft editing and revision. **Zebunnahar Yasmin**: molecular investigation. **Nachimuthu Senthil Kumar**: writing review, editing and revision. **Amam Zonaed Siddiki**: supervision, data analysis, writing review, editing and laboratory facilities. **Md. Farid Ahsan**: supervision, validation, management, writing, review and editing and laboratory facilities.

## Ethics Statement

The Institutional Animal Ethics Committee approved this work, which included collecting squirrel blood samples and extracting DNA. Licensed veterinarians drew blood samples from the squirrels following IACUC guidelines. All approaches were carried out under the ‘Regulations for Animal Experiments’ at Chattogram Veterinary and Animal Science University, with the university's specific attribute and ethical approval.

## Conflicts of Interest

The authors declare no conflicts of interest.

## Peer Review

The peer review history for this article is available at https://www.webofscience.com/api/gateway/wos/peer‐review/10.1002/vms3.70550.

## Data Availability

The sequence data have been submitted to the NCBI database under the accession numbers MT227570‐MT227575 and MT252006.
